# Procedural factors outweigh anatomical morphometry in predicting postoperative pain following retrograde intrarenal surgery

**DOI:** 10.1007/s00345-026-06354-9

**Published:** 2026-03-17

**Authors:** Mert Başaranoğlu, Ahmet Turhan, Ali Nebioğlu, Mesut Tek, Erdem Akbay

**Affiliations:** 1 Department of Urology, Faculty of Medicine, Mersin University, Mersin, Turkey; 2 Department of Urology, Mersin City Training and Research Hospital, Mersin, Turkey

**Keywords:** Retrograde intrarenal surgery, Postoperative pain, Anatomical morphometry, Gender differences, Double-J stent, Pain prediction

## Abstract

**Purpose:**

Postoperative pain affects 23–45% of RIRS patients, but predictive factors remain unclear. This study evaluated whether anatomical morphometric assessment predicts postoperative pain when controlling for procedural variables in patients with normal upper tract anatomy, and identified which factors most significantly influence pain after RIRS.

**Methods:**

This prospective single-center study included 420 consecutive patients undergoing RIRS (September 2024–May 2025). After exclusions, 320 patients completed three-week follow-up. Forty-five anatomical parameters and procedural variables (intrarenal pressure, laser parameters, access sheath use, complications) were recorded. Pain was assessed using VAS (seven timepoints) and Turkish USSQ. Analysis employed multiple comparison corrections, hierarchical regression, propensity score matching, and cross-validation.

**Results:**

Among 320 patients (67.8% male; mean age 51.6 ± 13.2 years), procedural factors primarily predicted postoperative pain. Intrarenal pressure (*r* = 0.448), complications (*r* = 0.418), and laser energy (*r* = 0.382) showed significant correlations with pain, accounting for most variance (R²=0.424). Anatomical parameters provided negligible predictive value (R²=-0.018), with no significant correlations after multiple testing correction. DJ stent pain was context-dependent, higher in high-pressure procedures but resolving rapidly post-removal in 89% of cases.

**Conclusion:**

Modifiable procedural factors—intrarenal pressure, complications, and laser energy—are the main determinants of postoperative pain following RIRS in patients with normal anatomy, while anatomical measurements lack predictive value. Optimizing intraoperative practices offers greater clinical benefit than preoperative morphometric assessment. These findings support deprioritizing resource-intensive anatomical profiling for pain prediction in RIRS. Preoperative anatomical profiling should not be routinely performed for pain prediction, as procedural optimization represents modifiable targets with greater impact on patient outcomes.

**Supplementary Information:**

The online version contains supplementary material available at 10.1007/s00345-026-06354-9.

## Introduction

Retrograde intrarenal surgery (RIRS) achieves stone-free rates exceeding 89% for 4–20 mm calculi with favorable safety profiles [1, 2]. However, postoperative pain affects 23–45% of patients beyond the immediate postoperative period [3]. Double-J (DJ) stent placement occurs in 70–90% of cases [1], yet the relative contribution of stents versus procedural factors to pain perception remains inadequately characterized. Postoperative pain represents a multifactorial phenomenon influenced by surgical trauma, inflammation, psychological factors, and potentially anatomical variations [3]. Previous attempts to predict RIRS pain outcomes have focused primarily on stone characteristics and stent-related factors, with inconsistent results and limited investigation of anatomical predictors. Morphometric assessment refers to the systematic quantification of anatomical dimensions using imaging modalities—in this context, CT-based measurements of pelvic and renal collecting system geometry to characterize individual anatomical variation. While morphometric assessment demonstrates value in obstetric procedures, its applicability to endourology remains unvalidated, and the hypothesis that pelvic morphometry would predict RIRS pain outcomes remained untested. We hypothesized that comprehensive pelvic-genital anatomical morphometry would demonstrate clinically meaningful correlations with postoperative pain outcomes when controlling for procedural factors, and that gender-specific anatomical differences would account for differential pain patterns. Specifically, we predicted narrower pelvic dimensions would correlate with higher pain scores; longer ureter length would associate with increased stent-related discomfort; and these anatomical predictors would enable morphometry-based pain prediction models with clinically useful accuracy (R²>0.25).

Gender-specific pain perception differences are well-established across surgical specialties, yet whether anatomical variations influence RIRS pain trajectories remains unexplored. Current literature lacks prospective investigations rigorously testing anatomical measurements while controlling for DJ stent placement, stone characteristics, and procedural variables. The absence of negative trials documenting anatomical prediction model limitations represents publication bias, potentially causing resource misallocation toward unvalidated protocols. Understanding which factors—anatomical versus procedural—most strongly predict pain has direct implications for resource allocation and clinical practice optimization. This prospective study assessed 420 consecutive RIRS patients with 320 completing the full protocol, systematically examining relationships between 45 anatomical parameters (37 pelvic, 8 renal) and three-week pain outcomes. Employing rigorous methodology including Bonferroni and Holm corrections, propensity score matching, and cross-validation, this investigation establishes whether anatomical morphometry provides clinically meaningful pain prediction when procedural factors are controlled.

## Materials and methods

This prospective single-center cohort study investigated relationships between pelvic-genital anatomical measurements and postoperative pain following RIRS. The protocol received approval from Mersin University Clinical Research Ethics Committee (2025/553, May 14, 2025) per Declaration of Helsinki. All participants provided written informed consent. Between September 2024 and May 2025, 420 consecutive adult patients scheduled for RIRS were initially assessed for eligibility. Inclusion criteria comprised age ≥ 18 years, renal/ureteral calculi 4–20 mm, available preoperative CT imaging, and ability to complete three-week follow-up. Exclusion criteria included urogenital anomalies (horseshoe kidney, ectopic kidney, ureteropelvic junction obstruction, duplex collecting systems, significant ureteral strictures), previous pelvic surgery, bilateral stones, active infection, pregnancy, chronic pain syndromes, opioid dependency, previous DJ stent placement, ASA > III, and psychiatric disorders interfering with pain assessment. After applying exclusion criteria (*n* = 68 excluded: 23 anatomical anomalies, 18 previous pelvic surgery, 12 bilateral stones, 8 active infection, 7 other criteria) and accounting for incomplete follow-up (*n* = 32 lost to follow-up or incomplete data), 320 patients were deemed eligible and completed the full three-week assessment protocol. Sample size calculation (G*Power 3.1.9.7) for correlation analysis with *r* = 0.3, α = 0.05, power = 90% yielded 112 patients required. Target enrollment of 320 patients accounted for multiple comparisons (45 anatomical parameters), anticipated attrition, and stratified analyses.

Anatomical measurements were obtained from preoperative CT scans (≤ 2 mm slice thickness, standardized protocols) independently by two blinded radiologists showing excellent inter-rater reliability (ICC: 0.92–0.98). Comprehensive assessment encompassed 45 anatomical parameters including 37 pelvic measurements (pelvic inlet anteroposterior/transverse diameters, mid-pelvis anteroposterior/transverse diameters, pelvic outlet anteroposterior/transverse diameters, pelvic depth, sacral slope, pelvic tilt, pelvic incidence, ureter length, upper/middle/lower ureter diameters, bladder capacity, bladder wall thickness, pelvic floor thickness, levator ani thickness, perineal body length, pubococcygeal distance, and gender-specific organ measurements) and 8 renal/stone-related parameters (kidney length/width/thickness, renal pelvis diameter, infundibulopelvic angle, calyceal neck diameter, parenchymal thickness, stone size). Complete parameter specifications are provided in Supplementary Table S1.

All procedures were performed under general anesthesia (endotracheal intubation, sevoflurane maintenance) to eliminate variability from spinal anesthesia and residual neuraxial blockade effects on early postoperative pain assessment. Standardized multimodal analgesia was administered intraoperatively: paracetamol 1 g IV 30 min before incision, dexketoprofen 50 mg IV after ureteral access, and fentanyl 1–2 µg/kg IV as needed during surgery. Postoperatively, patients received scheduled paracetamol 1 g every 6 h and dexketoprofen 50 mg every 12 h for 48 h, with tramadol 50-100 mg as rescue analgesia (VAS ≥ 4). This standardized protocol ensured consistent analgesic baseline across the cohort for valid pain score comparisons.

RIRS procedures used standardized techniques: semi-rigid then flexible ureteroscopy, holmium laser lithotripsy, systematic stone extraction. All procedures were performed by experienced urologists (> 200 RIRS procedures annually) using standardized protocols to minimize operator variability. Flexible ureteroscopy was performed using reusable Karl Storz Flex-X2S digital flexible ureteroscope (7.5 Fr working shaft diameter, 3.6 Fr working channel, Karl Storz Endoskope, Tuttlingen, Germany) in 245 cases (76.6%), and single-use Boston Scientific LithoVue digital flexible ureteroscope (8.5 Fr distal tip, 3.6 Fr working channel, Boston Scientific, Marlborough, MA, USA) in 75 cases (23.4%). Ureteral access sheaths when used were 11.5/13F (Cook Medical, Bloomington, IN, USA) or 12/14F (Boston Scientific) depending on ureteral caliber. The scope-to-sheath diameter ratio ranged from 0.58 to 0.65, allowing adequate irrigation outflow around the ureteroscope shaft. Intrarenal pressure was measured via a purpose-modified ureteral access sheath system incorporating a side-channel pressure transducer (Stryker T5 IntraCompartmental Pressure Monitor System, Stryker Corporation, Kalamazoo, MI, USA). The pressure sensor was positioned within the renal pelvis via the access sheath side-port, providing direct measurement of true intrarenal pressure rather than irrigation inflow pressure. Mean pressure was calculated as time-weighted average across the entire operative duration; peak pressure represented maximum recorded value during the procedure. Pressure measurements were recorded at 10-second intervals. All procedures utilized the Lumenis Pulse 120 H Holmium: YAG laser system. Moses Technology pulse modulation was employed in 187 procedures (58.4%), with remaining cases using standard pulse mode. Settings were individualized based on stone characteristics: typical parameters included pulse energy 0.5–1.2 J and frequency 10–25 Hz for dusting fragmentation, versus 1.0–2.0 J and frequency 5–15 Hz for fragmentation. Long pulse width mode was preferentially used for larger, harder stones (Hounsfield units > 1000), while short pulse mode was used for softer stones to minimize retropulsion. Irrigation was delivered using a standardized protocol combining gravity-based flow (saline bag at 100–150 cm height) with controlled manual supplementation via hand-pump bulb syringe when needed for visibility. Target irrigation pressure was < 150 mmHg at the pump level, consistent with international practice patterns. Automated irrigation systems were not used. Comprehensive intraoperative data were prospectively collected including total laser energy (kilojoules), total lasing time (minutes), ureteral access sheath usage, irrigation volume, ureteral manipulation (dilation degree, scope passages), and complications graded by standardized criteria (mucosal injuries grade 0–3, perforation, bleeding, stone migration). DJ stent placement was surgeon-discretion based on operative duration > 60 min, ureteral trauma, residual fragments, or solitary kidney. Stents (4.8 or 6.0 French, polyurethane or silicone) remained 21 ± 7 days. Final population: 275 stented (85.9%), 45 non-stented (14.1%).

Pain assessment utilized Visual Analog Scale (0–10) at 2 h, 6 h, 24 h, 48 h, 7d, 14d, 21d postoperatively, plus 24 h and 7d post-stent removal by blinded staff. Stent-related symptoms were evaluated using validated Turkish USSQ (six domains, 1–7 scale) at 21 days [4]. Quality of life and satisfaction used 10-point scales.

Statistical analyses utilized SPSS 28.0 and R 4.3.1. Data are presented as mean ± SD or median (IQR). Between-group comparisons employed t-tests or Mann-Whitney U tests; categorical variables employed chi-square or Fisher’s exact tests. Effect sizes were calculated as Cohen’s d. Correlation analyses utilized Pearson or Spearman coefficients based on data distribution. Given extensive multiple testing (315 tests: 45 anatomical parameters × 7 outcomes), Bonferroni correction controlled family-wise error rate (α = 0.000159; 0.05/315). While controlling Type I error, Bonferroni increases Type II error risk. Post-hoc power analysis demonstrated that *n* = 320 with α = 0.000159 provided 90% power for *r* ≥ 0.23 and 80% power for *r* ≥ 0.20. To address Bonferroni’s conservatism, Holm stepwise correction was applied as sensitivity analysis, maintaining family-wise error control with greater statistical power. For procedural variables (intrarenal pressure, laser, access sheath, complications), separate analyses employed appropriate correction (5 parameters × 7 outcomes = 35 tests, α = 0.00143).

Three hierarchical regression models compared anatomical versus procedural predictive value: Model 1 (Anatomical) included parameters with |r|>0.10; Model 2 (Procedural) included intrarenal pressure, laser energy, access sheath, and complications; Model 3 (Combined) integrated both sets to assess incremental value (ΔR²). Multicollinearity was assessed using variance inflation factors (VIF > 5); model performance was evaluated using R², adjusted R², RMSE, and AIC. Propensity score matching (1:1, caliper = 0.2) addressed stent confounding, matching on stone size, stone location, operative time, mean intrarenal pressure, laser energy, and patient age. Model stability was assessed via 10-fold cross-validation with 1000 bootstrap iterations. Fisher’s z-transformation compared correlation coefficients between genders.

## Results

**Fig. 1:**
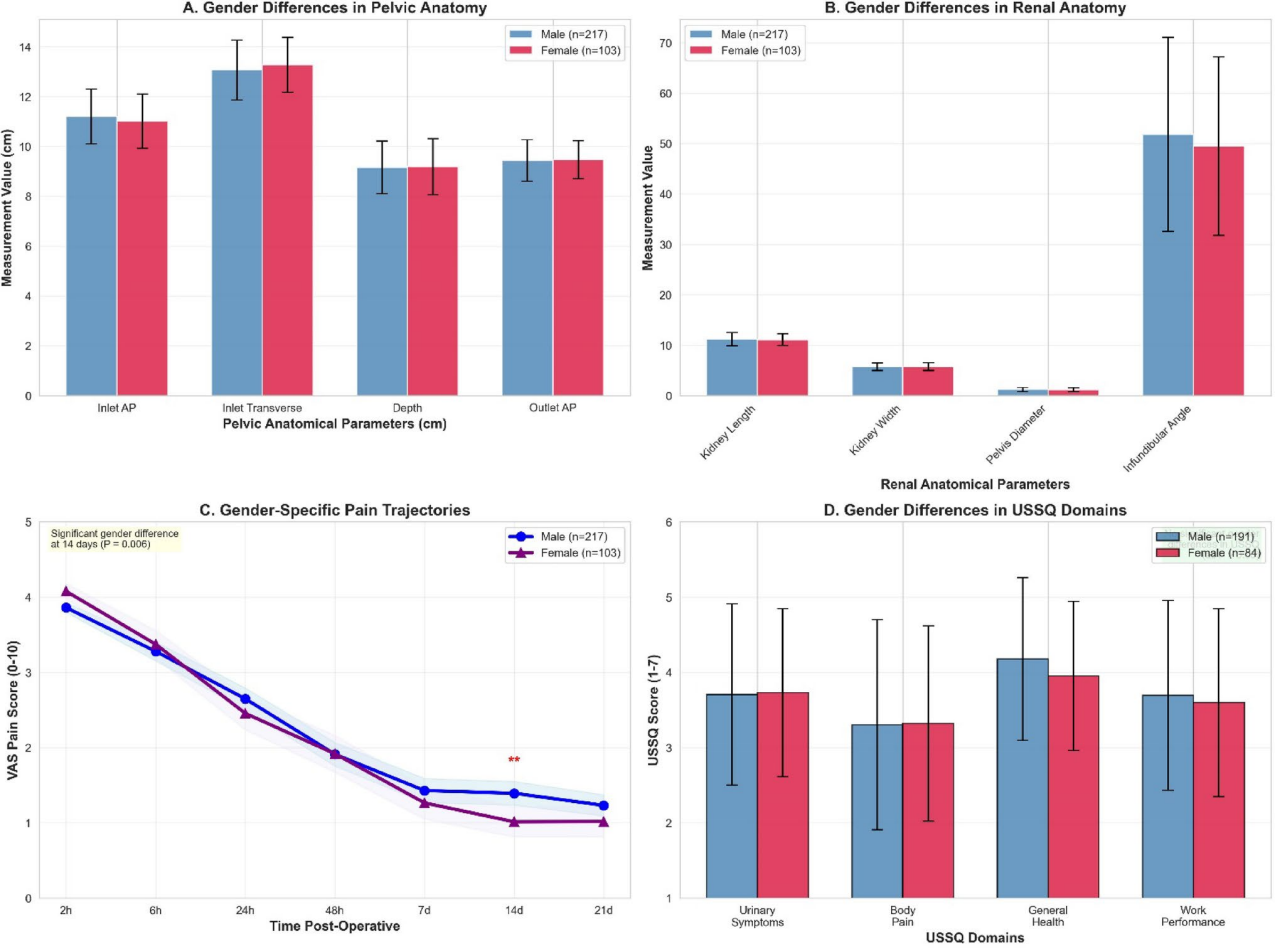
Gender-specific anatomical analysis and pain patterns. **A** Gender differences in key pelvic anatomical measurements. Bar graph compares pelvic anatomical parameters (pelvic inlet AP diameter, pelvic inlet transverse diameter, pelvic depth, pelvic outlet AP diameter) between male (blue, *n* = 217) and female (red, *n* = 103) patients. Error bars represent standard deviations. While trends toward gender differences are observed (males showing larger AP diameters, females larger transverse diameters), most differences do not reach statistical significance (all *P* > 0.05 except where indicated by asterisks). These subtle anatomical differences do not translate to clinically meaningful pain prediction capacity. **B** Gender Differences in Renal Anatomical Measurements. Bar graph presents renal anatomical parameters (kidney length, kidney width, pelvic diameter, infundibulopelvic angle) stratified by gender (blue = male, red = female). Error bars represent standard deviations. Gender differences in renal anatomy are minimal and not statistically significant (all *P* > 0.10), indicating similar renal collecting system configurations between sexes. Statistical significance indicated by asterisks where *P* < 0.05. **C** Gender-Specific Pain Trajectories Over Time. Line graph displays mean VAS pain scores over the three-week follow-up period separated by gender (blue line = male [*n* = 217], purple line = female [*n* = 103]). Shaded areas represent 95% confidence intervals for each gender group. Both genders demonstrate similar overall pain resolution patterns with overlapping trajectories at most time points. Significant gender difference emerged at 14-day assessment (male: 1.39 ± 1.18 vs. female: 1.01 ± 1.04, *P* = 0.006), indicated by asterisk. This time-specific difference suggests potential gender-based pain resolution patterns warranting further investigation. **D** Gender Differences in USSQ Domain Scores for Stented Patients. Bar graph compares USSQ symptom domain scores between male (blue, *n* = 191 stented males) and female (red, *n* = 84 stented females) patients. Domains assessed include urinary symptoms, body pain, general health, and work performance (all scored 1–7, lower = better). Error bars represent standard deviations. No statistically significant gender differences were detected across any USSQ domain (all *P* > 0.05), indicating consistent stent-related symptom burden patterns regardless of biological sex despite anatomical differences

**Fig. 2:**
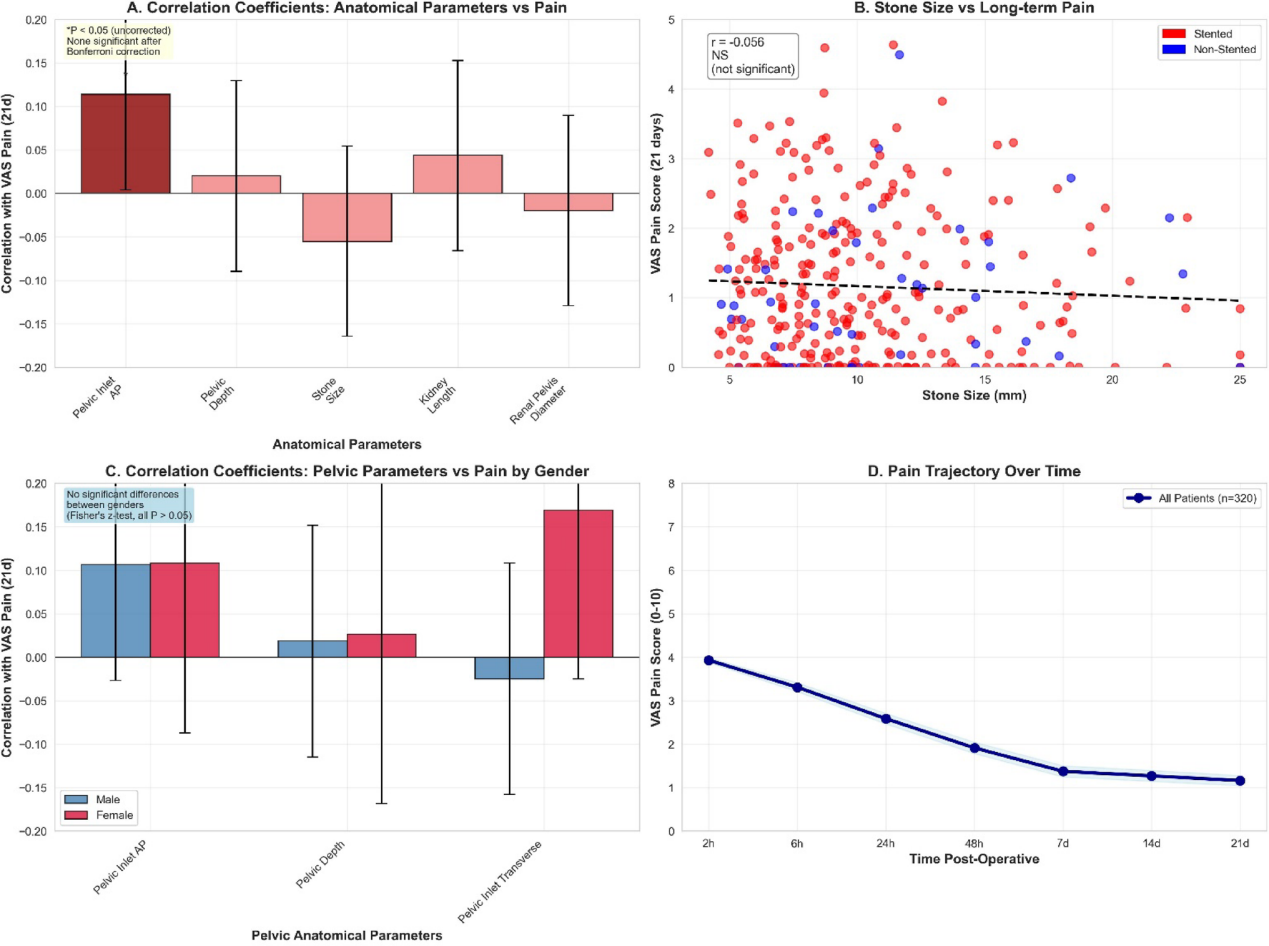
Anatomical measurements and pain correlation analysis. **A** Correlation coefficients between anatomical parameters and 21-day pain scores. Bar graph displays correlation coefficients (Pearson’s r) between key anatomical parameters (pelvic inlet anteroposterior diameter, pelvic depth, stone size, kidney length, and renal pelvis diameter) and 21-day VAS pain scores. Bars are colored according to uncorrected statistical significance levels (*P < 0.05 shown in dark red). All displayed correlations demonstrate weak effect sizes (all |r| < 0.15) and none maintain significance after Bonferroni correction (α = 0.000159). Error bars represent 95% confidence intervals. **B** Stone Size versus Long-Term Pain Outcomes Stratified by DJ Stent Status. Scatter plot displays the relationship between stone size (mm, x-axis) and 21-day VAS pain scores (y-axis). Data points are colored by DJ stent placement status (red = stented patients [*n* = 275], blue = non-stented patients [*n* = 45]). The dashed regression line indicates the weak overall inverse relationship (*r* = -0.056, *P* = 0.322), demonstrating the absence of clinically meaningful association. Similar scatter patterns between stented and non-stented groups visually confirm the lack of stent effect on pain-stone size relationships. **C** Gender-Specific Correlations Between Pelvic Anatomical Parameters and 21-Day Pain. Bar graph compares correlation coefficients between pelvic anatomical parameters (pelvic inlet AP diameter, pelvic depth, pelvic inlet transverse diameter) and 21-day VAS pain scores, stratified by gender (blue = male [*n* = 217], red = female [*n* = 103]). Both gender groups demonstrate comparable weak correlation patterns, with no statistically significant differences in correlation strengths between genders (Fisher’s z-tests, all *P* > 0.05). Error bars represent 95% confidence intervals. **D** Longitudinal Pain Trajectory Over Three-Week Follow-Up Period. Line graph illustrates mean VAS pain scores (y-axis) across seven assessment time points (x-axis: 2 h, 6 h, 24 h, 48 h, 7d, 14d, 21d postoperatively) for the entire study cohort (*n* = 320). Pain demonstrates consistent decline from initial postoperative values (3.93 ± 0.50) to 21-day assessment (1.16 ± 1.06), representing 70.5% improvement. The smooth trajectory curve indicates predictable pain resolution patterns consistent with published RIRS literature. Shaded area represents 95% confidence interval

The final study cohort comprised 320 patients who completed the full three-week assessment protocol (Table 1). The population included 217 males (67.8%) and 103 females (32.2%) with mean age 51.6 ± 13.2 years. DJ stent placement occurred in 275 patients (85.9%), mean operative time was 62.9 ± 17.0 min, and stone-free rate was 90.0%. Complications (Clavien-Dindo classification) occurred in 55 patients (17.2%): Grade I included postoperative fever > 38 °C without positive cultures (*n* = 18, 5.6%) and hematuria requiring prolonged observation (*n* = 12, 3.8%); Grade II included urinary tract infection requiring antibiotics (*n* = 18, 5.6%); Grade III included ureteral perforation requiring prolonged stenting (*n* = 5, 1.6%) and clot retention requiring cystoscopy with clot evacuation (*n* = 2, 0.6%). Infectious complications (fever and UTI, *n* = 36, 11.2%) correlated significantly with mean intrarenal pressure (*r* = 0.387, *P* < 0.001), supporting the mechanistic link between elevated pressure, pyelovenous backflow, and infectious sequelae. Mechanical complications (perforation, bleeding, *n* = 19, 5.9%) showed stronger correlation with total laser energy (*r* = 0.412, *P* < 0.001) and access sheath usage (*r* = 0.315, *P* < 0.001), consistent with trauma-related etiology. Comprehensive anatomical measurements stratified by gender are detailed in Table 2. Significant gender differences emerged in ureter length (male 25.62 ± 3.39 vs. female 24.25 ± 2.90 cm, *P* < 0.001, d = 0.42) and bladder capacity (female 443.13 ± 81.70 vs. male 411.31 ± 83.37 mL, *P* = 0.001, d=-0.38), while other pelvic dimensions showed minimal differences (all *P* > 0.05; Fig. 1A, B). Pain intensity demonstrated 70.5% reduction from 3.93 ± 0.50 at 2 h to 1.16 ± 1.06 at 21 days (Table 2; Fig. 2D).

Intraoperative factors demonstrated strong associations with postoperative pain (Table 3). Mean intrarenal pressure averaged 38.5 ± 12.3 mmHg (range 15–85), correlating significantly with 21-day pain (*r* = 0.448, *P* < 0.001, 20.1% variance). Patients in the highest pressure quartile (> 45 mmHg) experienced markedly elevated pain compared to the lowest quartile (2.12 ± 1.23 vs. 0.68 ± 0.78, *P* < 0.001, d = 1.35). Total laser energy averaged 8.7 ± 6.2 kJ (range 0.5–32.4), demonstrating moderate correlation with pain (*r* = 0.382, *P* < 0.001, 14.6% variance). Access sheath usage (*n* = 198, 61.9%) associated with higher pain versus no-sheath procedures (21-day VAS 1.38 ± 1.15 vs. 0.82 ± 0.84, *P* = 0.003, d = 0.53). Intraoperative complications (mucosal injury grade ≥ 2) in 47 patients (14.7%) strongly predicted elevated pain (2.45 ± 1.35 vs. 1.02 ± 0.94, *P* < 0.001, d = 1.21). Both Bonferroni (α = 0.00143 for 35 comparisons) and Holm corrections confirmed significance for intrarenal pressure, laser energy, and complications (all *P* < 0.001), but not access sheath usage (Bonferroni *P* = 0.003, Holm *P* = 0.005, both NS after correction).

Initial analysis revealed no significant pain differences between stented and non-stented patients during the indwelling period (all *P* > 0.05). However, subgroup analysis uncovered important procedural context-dependency: among high intrarenal pressure procedures (> 45 mmHg), stented patients experienced significantly elevated pain compared to non-stented controls (21-day VAS 2.45 ± 1.28 vs. 1.12 ± 0.95, *P* = 0.008), whereas low-pressure procedures (< 30 mmHg) showed minimal stent impact (0.89 ± 0.82 vs. 0.71 ± 0.76, *P* = 0.412). Propensity score matching (*n* = 45 pairs) confirmed absence of overall main stent effects (all *P* > 0.10), likely reflecting small non-stented sample size and selection bias toward uncomplicated procedures. Post-stent removal, 89.1% achieved complete pain resolution within 48 h, confirming stents as significant pain contributors whose effects varied by procedural context. USSQ analysis in stented patients (*n* = 275) demonstrated moderate symptom burden (total score 21.98 ± 3.17) with high quality of life (9.07 ± 1.00) and satisfaction (9.05 ± 1.08). Gender-stratified USSQ showed no significant differences (all *P* > 0.05; Fig. 1D).

Stone-free status correlation with pain outcomes: Among 288 stone-free patients (90.0%) versus 32 patients with residual fragments, 21-day VAS pain scores showed no significant difference (1.14 ± 1.05 vs. 1.28 ± 1.13, *P* = 0.442, d = 0.13). Similarly, residual fragment status did not correlate with 7-day pain (*r* = 0.067, *P* = 0.234), USSQ total score (*r* = 0.089, *P* = 0.127), or quality of life (*r*=-0.058, *P* = 0.302). However, patients with residual fragments had significantly longer operative times (71.3 ± 19.8 vs. 61.5 ± 16.2 min, *P* = 0.003), higher total laser energy (11.8 ± 7.4 vs. 8.3 ± 5.9 kJ, *P* = 0.006), and elevated mean intrarenal pressure (44.2 ± 13.5 vs. 37.6 ± 11.9 mmHg, *P* = 0.004). These findings suggest residual fragments serve as a marker for procedurally complex cases rather than an independent pain predictor; the procedural variables themselves (pressure, energy, duration) mediate the relationship between stone burden and pain outcomes.

Correlation analysis revealed only weak associations between anatomical parameters and pain outcomes (Table 4; Fig. 2A-C). The strongest correlation was ureter length versus satisfaction (*r* = 0.172, 3.0% variance). After Bonferroni correction (α = 0.000159 for 315 tests), no correlations maintained significance. Sensitivity analysis using Holm correction similarly yielded no significant correlations, confirming negative findings were not artifacts of overly conservative correction. Gender-specific analyses showed comparable weak patterns in both sexes (Fisher’s z-test, all *P* > 0.05), with no clinically meaningful correlations (|r|>0.3) identified. Hierarchical regression compared anatomical versus procedural predictive value for 21-day pain. Model 1 (Anatomical) included parameters with |r|>0.10, achieving R²=0.029, adjusted R²=-0.018, indicating negligible predictive capacity (VIF < 2.1). Model 2 (Procedural) included intrarenal pressure, laser energy, access sheath usage, and complications, demonstrating substantial predictive value (R²=0.424, adjusted R²=0.417, RMSE = 0.82 ± 0.11, AIC = 892.3). Intrarenal pressure emerged as strongest predictor (β = 0.368, *P* < 0.001), followed by complications (β = 0.285, *P* < 0.001) and laser energy (β = 0.197, *P* = 0.002). Model 3 (Combined) yielded R²=0.451, adjusted R²=0.437, RMSE = 0.80 ± 0.10, AIC = 887.1, with anatomical parameters contributing minimal incremental value (ΔR²=0.027, *P* = 0.082). Cross-validation (10-fold, 1000 bootstrap iterations) confirmed Model 2’s superiority over Model 1 (RMSE 0.82 ± 0.11 vs. 1.08 ± 0.15, *P* < 0.001), while Model 3 showed only marginal improvement over Model 2 (RMSE 0.80 ± 0.10 vs. 0.82 ± 0.11, *P* = 0.214), confirming procedural factors as primary pain determinants with minimal incremental value from anatomical morphometry. DJ stent characteristics (diameter, material) and duration showed no significant associations with pain outcomes (all |r|<0.04, all *P* > 0.05).

## Discussion

This prospective study of 320 RIRS patients establishes procedural factors—intrarenal pressure (*r* = 0.448), complications (*r* = 0.418), and laser energy (*r* = 0.382)—as primary pain determinants, while anatomical morphometry contributes negligible value (R²=-0.018) in patients with normal upper tract anatomy. Hierarchical regression demonstrated procedural variables achieved R²=0.424, whereas combined models incorporating anatomical parameters yielded only ΔR²=0.027 (*P* = 0.082). Our findings align with contemporary RIRS literature reporting stone-free rates exceeding 89% [5], yet systematically quantify relative contributions of anatomical versus procedural factors. The observed 70.5% pain reduction from 3.93 ± 0.50 (2 h) to 1.16 ± 1.06 (21d) mirrors temporal patterns in RIRS cohorts [6, 7].

Among these procedural variables, intrarenal pressure emerged as the strongest pain predictor, consistent with mechanistic studies demonstrating pyelovenous backflow at 13.6–27.2 cmH₂O and tissue injury above 60 mmHg [8, 9]. Patients in highest pressure quartile (> 45 mmHg) experienced markedly elevated pain versus lowest quartile (2.12 ± 1.23 vs. 0.68 ± 0.78, d = 1.35). Elevated intrarenal pressure correlates with infectious complications and renal damage [10, 11], with cumulative exposure potentially predicting risk better than peak values [12]. Our protocol targeted < 30 mmHg, yet 25% exceeded 45 mmHg, indicating need for intelligent pressure-control platforms [13]. High-pressure procedures with stents showed elevated pain (2.45 ± 1.28 in > 45 mmHg vs. 0.89 ± 0.82 in < 30 mmHg, *P* = 0.008), establishing pressure-dependent stent morbidity—a relationship inadequately explored in DJ stent literature focusing on stent design over procedural context [14, 15].

These findings have immediate clinical implications. First, routine intrarenal pressure monitoring should be implemented as standard practice, with protocols targeting < 30 mmHg and interventions when pressures exceed 40 mmHg. Second, laser energy optimization through newer technologies—Moses pulse modulation, thulium fiber laser, or high-power holmium systems—can reduce total energy delivery while maintaining stone-free rates. Third, meticulous surgical technique minimizing mucosal injury represents a modifiable target with substantial impact on pain outcomes. Fourth, suction-enabled ureteral access sheaths simultaneously improve stone clearance and pressure control. In contrast, preoperative morphometric assessment of pelvic anatomy provides negligible predictive value and represents an inefficient allocation of imaging analysis resources.

The relationship between DJ stents and postoperative pain demonstrated important context-dependency. We emphasize that this study was not designed as a randomized trial comparing stent versus non-stent protocols. Initial analysis revealed no overall difference between stented versus non-stented patients (*P* > 0.05), yet 89.1% achieved pain resolution within 48 h post-removal. These findings are reconciled by three factors. First, inadequate statistical power in small non-stented cohort (*n* = 45) with selection bias toward uncomplicated procedures limits comparative inference. Second, subgroup analysis uncovered critical procedural context-dependency: high-pressure procedures (> 45 mmHg) with stents demonstrated significantly elevated pain versus non-stented controls (2.45 ± 1.28 vs. 1.12 ± 0.95, *P* = 0.008), whereas low-pressure procedures (< 30 mmHg) showed minimal stent impact (0.89 ± 0.82 vs. 0.71 ± 0.76, *P* = 0.412). This pressure-pain interaction has not been systematically evaluated in randomized stent trials [16, 17]. Third, rapid post-removal resolution confirms stents contribute substantially to pain burden. Our USSQ score (21.98 ± 3.17) falls within favorable range compared to published values (25–35) [18], with domain scores aligning with Turkish validation study [4]. Recent trials demonstrate pharmacological interventions (theophylline, mirabegron, alpha-blockers) modestly reduce stent-related symptoms [16, 19], yet our findings suggest procedural optimization (pressure control, complication prevention) may offer more substantial symptom mitigation than pharmacotherapy.

While stent effects varied by procedural context, gender-specific anatomical differences showed distinct patterns. Gender differences emerged in ureter length (male 25.62 ± 3.39 vs. female 24.25 ± 2.90 cm, *P* < 0.001) and bladder capacity (*P* = 0.001), consistent with anatomical sex differences [20]. However, these variations failed to predict pain, whereas 14-day pain showed gender difference (male 1.39 ± 1.18 vs. female 1.01 ± 1.04, *P* = 0.006, d = 0.33). This suggests biological sex—encompassing hormonal and neurophysiological factors—serves as superior stratification variable than isolated anatomical measurements. The finding contradicts hypotheses predicting pelvic dimensions would correlate with pain, yet al.igns with broader literature emphasizing biopsychosocial determinants [21]. Our 45-parameter anatomical assessment represents the most extensive morphometric evaluation in endourological literature, yet maximum correlation (*r* = 0.172) explained only 3.0% variance—below clinically useful thresholds. This challenges personalized medicine proposals advocating morphometric profiling, redirecting focus toward modifiable procedural parameters. Comprehensive preoperative morphometric assessment requires specialized imaging analysis and radiologist time, whereas intraoperative pressure monitoring represents readily implementable interventions with greater impact.

Laser energy demonstrated moderate correlation with pain (*r* = 0.382, 14.6% variance), consistent with energy as surrogate marker for stone complexity [22]. Recent advances—Moses modulation, thulium fiber laser, high-power systems—achieve faster fragmentation with reduced thermal injury [23, 24]. Thulium fiber laser reduces lasing time and total energy versus holmium: YAG for equivalent stone-free rates [22]. Access sheath usage (61.9%) associated with higher pain (1.38 ± 1.15 vs. 0.82 ± 0.84, *P* = 0.003), though significance diminished after correction. This appears contradictory to data showing sheath reduces complications [25, 26], yet reflects indication bias: larger stones requiring sheaths associate with greater trauma. Propensity-matched analyses confirm sheath improves outcomes controlling for stone burden [27, 28]. Suction-enabled sheaths simultaneously improve clearance while controlling pressure [29].

While no anatomical correlations survived Bonferroni correction (α = 0.000159), convergent evidence supports genuine absence of associations rather than Type II error. First, maximum correlation (*r* = 0.172) explained only 3.0% variance. Second, *n* = 320 provided 90% power to detect *r* ≥ 0.156, yet uncorrected analyses revealed no correlations exceeding *r* = 0.18. Third, Holm correction yielded identical conclusions. Fourth, hierarchical regression achieved negative adjusted R²=-0.018. Fifth, cross-validation showed prediction accuracy equivalent to chance (RMSE 1.08 ± 0.15 vs. null 1.06 ± 0.14, *P* = 0.523). We acknowledge Bonferroni increases Type II error risk; however, clinical utility remains questionable given observed effect magnitudes. Pain perception involves complex biopsychosocial mechanisms—genetic polymorphisms, psychological factors, central sensitization [30]—inadequately captured by static anatomical morphometry. Our findings align with absence of robust anatomical predictors in RIRS literature [5], suggesting publication bias may have suppressed prior negative trials.

This investigation utilized prospective consecutive enrollment of 320 patients with comprehensive 45-parameter anatomical assessment, validated outcomes (Turkish USSQ [4], seven-timepoint VAS), rigorous corrections (Bonferroni, Holm, propensity matching, cross-validation), and blinded radiologist assessments (ICC 0.92–0.98). However, several limitations require consideration.

We acknowledge that exclusion of anatomical anomalies (*n* = 23) represents an important limitation that restricts generalizability. Our findings apply specifically to patients with normal upper tract anatomy, where morphometric variation within the physiological range does not predict pain outcomes. In complex anatomical cases—horseshoe kidney, ectopic kidney, marked collecting system abnormalities—anatomical factors likely exert greater influence on procedural difficulty and complications, and thus indirectly impact pain. The restriction of range introduced by excluding these cases was necessary to test our primary hypothesis regarding physiological morphometric variation, but precludes extrapolation to anatomically complex cases where individualized surgical planning based on anatomy remains essential.

Similarly, exclusion of patients with previous DJ stent placement (common in staged procedures) limits generalizability. Pre-stenting induces passive ureteral dilation, potentially reducing intrarenal pressure during subsequent RIRS and facilitating ureteral access sheath placement. Our cohort therefore represents primary procedures without pre-dilation, potentially overestimating absolute pressure values and access-related trauma compared to staged approaches. However, this strengthens rather than weakens our core finding: even in this ‘worst-case’ scenario for pressure and access difficulty, anatomical morphometry failed to predict pain outcomes. In pre-stented populations where procedural factors may be further attenuated, anatomical predictors would likely show even weaker associations. Nevertheless, dedicated studies in pre-stented cohorts would clarify whether the relative weight of procedural versus anatomical factors differs in this subgroup. The single-center Turkish population limits generalizability; multicenter validation is needed. Three-week follow-up captures acute/subacute but not chronic pain (5–10% of stented patients) [31]. Absence of psychological, genetic (COMT, OPRM1, SCN9A), and inflammatory biomarker assessment precludes evaluation of factors potentially explaining more variance. Our stent duration (21 ± 7 days) exceeds North American practice (7–14 days) [32], potentially limiting generalizability, though reviews report duration minimally impacts symptoms [33]. High stent rate (85.9%) with small unstented cohort (*n* = 45) limits natural pain history characterization. Static CT cannot capture dynamic characteristics (peristalsis, contractility, pelvic floor function). While morphometric profiling is not standard practice, personalized medicine interest warranted investigation. Our negative findings redirect resources toward procedural optimization rather than morphometric profiling, though biological sex may inform counseling. Future research should prioritize biopsychosocial approaches incorporating genetic screening, psychological tools, inflammatory biomarkers, and machine learning [34]. Randomized trials isolating DJ stent effects while controlling procedural factors would clarify stent-pain mechanisms. Intraoperative decision-support tools integrating real-time pressure monitoring and predictive algorithms may optimize outcomes [35, 36].

**Table 1:** Patient demographics and baseline characteristics

Characteristic	Value	Percentage (%)
Total patients, n	320	100.0
Age, years (mean ± SD)	51.6 ± 13.2	Range: 18–85
Gender, n (%)		
- Male	217	67.8
- Female	103	32.2
Body mass ındex, kg/m²	27.0 ± 4.1	
Stone size, mm (mean ± SD)	10.3 ± 4.3	
Stone location, n (%)		
- Renal pelvis	108	33.8
- Lower calyx	71	22.2
- Upper calyx	67	20.9
- Middle calyx	48	15.0
- Ureter	26	8.1
Stone densİty, HU	849 ± 250	
DJ stent placement, n (%)		
- Stent placed	275	85.9
- No stent	45	14.1
Stent size (Fr), n (%)ᵃ		
− 4.8 Fr	245	89.1
− 6.0 Fr	30	10.9
Ureteral access sheath, n (%)		
- UAS used	198	61.9
- No UAS	122	38.1
Operative time, min (mean ± SD)	62.9 ± 17.0	
Stone-free rate, n (%)	288	90.0
Complications (Clavien-Dindo), n (%)		
- None	265	82.8
- Grade 1	30	9.4
- Grade 2	18	5.6
- Grade 3	7	2.2

Demographic and clinical characteristics of the 320 patients who completed the three-week postoperative assessment protocol following retrograde intrarenal surgery (September 2024–May 2025)

ᵃAmong patients with DJ stent placement (*n* = 275)

SD, standard deviation; HU, Hounsfield units; Fr, French; UAS, ureteral access sheath

**Table 2:** Anatomical measurements and pain outcomes

Parameter	Overall (*n* = 320)	Male (*n* = 217)	Female (*n* = 103)	*P*-valueᵃ	Effect sizeᵇ
Key anatomical measurements					
Pelvic Inlet AP diameter, cm	11.14 ± 1.10	11.20 ± 1.10	11.02 ± 1.09	0.162	0.17
Ureter length, cm	25.18 ± 3.30	25.62 ± 3.39	24.25 ± 2.90	< 0.001***	0.42
Bladder capacity, mL	421.55 ± 84.04	411.31 ± 83.37	443.13 ± 81.70	0.001**	-0.38
Kidney length, cm	11.16 ± 1.26	11.19 ± 1.31	11.09 ± 1.16	0.512	0.08
Pain scores (VAS 0–10)					
2 h post-operative	3.93 ± 0.50	3.95 ± 0.48	3.89 ± 0.54	0.312	0.12
6 h post-operative	3.31 ± 0.95	3.35 ± 0.96	3.22 ± 0.92	0.234	0.14
24 h post-operative	2.59 ± 1.10	2.64 ± 1.12	2.50 ± 1.05	0.287	0.13
48 h post-operative	1.91 ± 1.21	1.95 ± 1.22	1.82 ± 1.18	0.356	0.11
7 days post-operative	1.38 ± 1.18	1.42 ± 1.20	1.30 ± 1.13	0.378	0.10
14 days post-operative	1.27 ± 1.15	1.39 ± 1.18	1.01 ± 1.04	0.006**	0.33
21 days post-operative	1.16 ± 1.06	1.19 ± 1.07	1.10 ± 1.04	0.456	0.08
USSQ domain scores (stented pts)					
Urinary symptoms (1–7)	3.71 ± 1.18	3.68 ± 1.16	3.78 ± 1.23	0.512	-0.08
Body pain (1–7)	3.31 ± 1.36	3.28 ± 1.34	3.38 ± 1.41	0.523	-0.07
Total USSQ score (6–42)	21.98 ± 3.17	21.89 ± 3.12	22.18 ± 3.28	0.456	-0.09
Quality of life outcomes					
Quality of life (1–10)	9.07 ± 1.00	9.05 ± 1.01	9.12 ± 0.98	0.523	-0.07
Patient satisfaction (1–10)	9.05 ± 1.08	9.01 ± 1.09	9.14 ± 1.06	0.287	-0.12

Key anatomical measurements and longitudinal pain assessment outcomes stratified by gender. Data include selected anatomical parameters, Visual Analog Scale (VAS) pain scores at seven timepoints, Ureteral Stent Symptoms Questionnaire (USSQ) domains, and quality of life metrics.Data: mean ± SD

ᵃIndependent t-tests (male vs. female)

ᵇCohen’s d (0.2 = small, 0.5 = medium, 0.8 = large)

*P < 0.05, **P < 0.01, ***P < 0.001

AP, anteroposterior; VAS, Visual Analog Scale; USSQ, Ureteral Stent Symptoms Questionnaire; SD, standard deviation

**Table 3:** Procedural factors and pain outcomes

Procedural variable	*N*	VAS pain (21d)	VAS pain (7d)	USSQ total	QoL (3 wks)	Patient satisfaction	Bonferroni sig.ᵃ	Holm sig.ᵇ
Intrarenal pressure								
Mean PRESSURE (mmHg)	320	0.448***	0.412***	0.385***	-0.398***	-0.425***	*P* < 0.001	*P* < 0.001
Peak pressure (mmHg)	320	0.401***	0.378***	0.356***	-0.362***	-0.381***	*P* < 0.001	*P* < 0.001
Laser parameters								
Total energy (kJ)	320	0.382***	0.349***	0.318***	-0.285***	-0.298***	*P* < 0.001	*P* < 0.001
Total lasing time (min)	320	0.328***	0.301***	0.286***	-0.257**	-0.271**	*P* < 0.001	*P* < 0.001
Mean power (W)	320	0.156*	0.142*	0.128*	-0.118	-0.135*	NS	NS
Access sheath								
UAS usage (yes/no)	320	0.289**	0.267**	0.245**	-0.221**	-0.238**	NS (*P* = 0.003)	NS (*P* = 0.005)
UAS size (F)	198	0.187*	0.165*	0.152*	-0.144*	-0.159*	NS	NS
Intraoperative factors								
Complication grade	320	0.418***	0.395***	0.372***	-0.348***	-0.365***	*P* < 0.001	*P* < 0.001
Operative time (min)	320	0.245**	0.228**	0.212**	-0.198**	-0.215**	NS	NS
Irrigation volume (L)	320	0.318***	0.295***	0.278***	-0.251**	-0.268**	*P* < 0.001	*P* < 0.001
N. scope passages	320	0.276**	0.258**	0.241**	-0.225**	-0.242**	NS	NS
Comparison to anatomy								
Strongest anatomicalᶜ	320	0.172*	0.115*	-0.096	-0.178**	0.172*	NS	NS

Correlation coefficients between intraoperative procedural variables and postoperative pain outcomes. Values represent Pearson or Spearman correlation coefficients (r) between procedural parameters and five outcome measures assessed at 21 days postoperatively or during stent indwelling period

*P < 0.05, **P < 0.01, ***P < 0.001 (uncorrected)

ᵃBonferroni correction: α = 0.00143 (35 comparisons)

ᵇHolm stepwise correction

ᶜStrongest anatomical correlation: ureter length vs. satisfaction (*r* = 0.172)

NS, not significant after correction

VAS, Visual Analog Scale; USSQ, Ureteral Stent Symptoms Questionnaire; QoL, quality of life; UAS, ureteral access sheath

**Table 4:** Correlation matrix - selected anatomical parameters vs. pain outcomes

Anatomical parameter	*N*	VAS pain (21D)	VAS pain (7d)	USSQ total score	QoL (3 wks)	Patient satisfaction	Bonferroni significance
Pelvic parameters							
Pelvic ınlet AP diameter	320	0.114*	0.009	-0.035	0.001	-0.050	NS
Pelvic outlet AP diameter	320	0.047	0.054	-0.049	-0.178**	0.110*	NS
Pelvic depth	320	0.020	0.023	0.014	-0.063	0.043	NS
Pelvic tilt (degrees)	320	-0.090	0.007	0.074	-0.001	0.128*	NS
Pelvic floor thickness	320	0.144*	0.093	-0.077	-0.171**	-0.011	NS
Renal parameters							
Kidney LENGTH	320	0.044	-0.088	-0.007	-0.032	0.015	NS
Kidney WİDTH	320	0.026	0.107	0.046	0.030	-0.039	NS
Renal pelvis diameter	320	-0.020	0.004	-0.066	-0.014	0.042	NS
Infundibulopelvic angle	320	0.017	-0.014	-0.035	0.019	-0.052	NS
Urinary tract parameters							
Ureter length	320	-0.049	0.029	-0.021	0.045	0.172**	NS
Upper ureter diameter	320	-0.070	0.011	-0.064	0.013	0.032	NS
Lower ureter diameter	320	0.084	0.067	-0.053	-0.062	0.104	NS
Bladder capacity	320	0.026	0.049	-0.096	-0.048	0.015	NS
Stone-related parameters							
Stone sizeᶜ	320	-0.056	-0.152**	0.012	0.001	0.026	NS

Representative anatomical parameters from comprehensive 45-parameter morphometric assessment showing correlation coefficients with postoperative pain outcomes. This table presents selected parameters from each anatomical category; complete data for all 45 measurements are available in Supplementary Table S1

*P < 0.05, **P < 0.01, ***P < 0.001 (uncorrected)

ᵃBonferroni correction: α = 0.000159 (315 comparisons: 45 parameters × 7 outcomes)

ᵇHolm stepwise correction; results identical to Bonferroni

ᶜStone size included in 45 parameters. Stone density excluded (material property, not morphometry)

NS, not significant after correction. *N* = 320; USSQ analysis *n* = 275 (stented patients)

Complete data for all 45 parameters in Supplementary Table S1

VAS, Visual Analog Scale; USSQ, Ureteral Stent Symptoms Questionnaire; QoL, quality of life; AP, anteroposterior; HU, Hounsfield units

## Conclusion

In patients with normal upper tract anatomy, procedural factors—intrarenal pressure, complications, and laser energy—rather than anatomical morphometry are the primary determinants of postoperative pain following RIRS. Hierarchical regression confirmed procedural variables provided substantial predictive value while anatomical parameters contributed negligibly, with minimal incremental benefit from combined models. Clinical implications favor implementing intrarenal pressure monitoring with defined thresholds, laser optimization strategies, and complication prevention protocols over resource-intensive morphometric assessment for pain prediction. Preoperative anatomical profiling should not be routinely performed for pain prediction in RIRS patients with normal anatomy, as procedural optimization represents modifiable targets with greater impact on patient outcomes. These findings apply specifically to patients with normal upper tract anatomy and should not be extrapolated to cases with significant anatomical anomalies, where individualized anatomical assessment remains important for surgical planning.

## Supplementary Information

Below is the link to the electronic supplementary material.

Supplementary Material 1

## Data Availability

Datasets generated during this study are available from the corresponding author upon reasonable request and with appropriate ethical approvals. Analysis code is available from the corresponding author upon reasonable request.
