# Supplementary material for: Procedural factors outweigh anatomical morphometry in predicting postoperative pain following retrograde intrarenal surgery
Source: World J Urol. 2026 Mar 17;44(1):247. doi: 10.1007/s00345-026-06354-9 (PMC12996379; doi:10.1007/s00345-026-06354-9)
Supplement: Supplementary file 1 — Supplementary Material 1 [file 345_2026_6354_MOESM1_ESM.docx]

# Supplementary Materials

## Supplementary Table S1: Complete Anatomical Parameter Measurements and Correlation with Pain Outcomes

**Study:** Procedural Factors Outweigh Anatomical Morphometry in Predicting Postoperative Pain Following RIRS in Patients with Normal Upper Tract Anatomy

This supplementary table provides comprehensive specifications for all 45 anatomical parameters assessed in this study, including mean values stratified by gender with statistical comparisons and individual correlation coefficients for all pain outcomes.

### Legend:

- **n=320 total patients** (217 males, 103 females)
- **VAS:** Visual Analog Scale (0-10)
- **USSQ:** Ureteral Stent Symptoms Questionnaire total score
- **r:** Pearson or Spearman correlation coefficient
- **Bonferroni correction:** α=0.000159 (0.05/315 comparisons)
- **Holm correction:** Stepwise method
- **P-values:** For gender differences (independent t-test or Mann-Whitney U test)
- ***P<0.05, **P<0.01, ***P<0.001** (uncorrected)
- **NS:** Not significant after correction

### A. PELVIC PARAMETERS (37 parameters)

#### A1. Pelvic Dimensions (12 parameters)

| Parameter | Unit | Male Mean±SD | Female Mean±SD | P-value | Cohen's d | VAS 21d (r) | VAS 7d (r) | USSQ (r) | QoL (r) | Satisfaction (r) | Bonferroni | Holm |
| --- | --- | --- | --- | --- | --- | --- | --- | --- | --- | --- | --- | --- |
| Pelvic Inlet AP Diameter | cm | 11.20±1.10 | 11.02±1.09 | 0.162 | 0.17 | 0.114* | 0.009 | -0.035 | 0.001 | -0.050 | NS | NS |
| Pelvic Inlet Transverse Diameter | cm | 12.85±0.95 | 13.12±0.89 | 0.012* | 0.29 | -0.082 | -0.045 | 0.023 | -0.089 | -0.012 | NS | NS |
| Pelvic Mid-plane AP Diameter | cm | 11.50±1.05 | 11.38±1.02 | 0.312 | 0.12 | 0.067 | 0.034 | -0.012 | 0.045 | 0.028 | NS | NS |
| Pelvic Mid-plane Transverse Diameter | cm | 10.25±0.88 | 10.45±0.85 | 0.056 | 0.23 | -0.045 | -0.023 | 0.015 | -0.034 | -0.018 | NS | NS |
| Pelvic Outlet AP Diameter | cm | 11.85±1.15 | 12.05±1.12 | 0.145 | 0.18 | 0.047 | 0.054 | -0.049 | -0.178** | 0.110* | NS | NS |
| Pelvic Outlet Transverse Diameter | cm | 10.15±0.92 | 10.68±0.88 | <0.001*** | 0.59 | -0.089 | -0.067 | 0.034 | -0.045 | -0.023 | NS | NS |
| Pelvic Depth | cm | 12.45±1.25 | 11.85±1.18 | <0.001*** | 0.49 | 0.020 | 0.023 | 0.014 | -0.063 | 0.043 | NS | NS |
| Interpubic Distance | cm | 9.85±0.75 | 10.25±0.72 | <0.001*** | 0.54 | -0.056 | -0.034 | 0.018 | -0.028 | -0.015 | NS | NS |
| Interspinous Distance | cm | 10.45±0.82 | 11.15±0.79 | <0.001*** | 0.87 | -0.078 | -0.056 | 0.023 | -0.045 | -0.029 | NS | NS |
| Intertuberous Distance | cm | 11.25±0.88 | 12.05±0.85 | <0.001*** | 0.93 | -0.092 | -0.068 | 0.034 | -0.056 | -0.038 | NS | NS |
| Diagonal Conjugate | cm | 12.65±1.05 | 12.95±1.02 | 0.018* | 0.29 | 0.045 | 0.028 | -0.015 | 0.034 | 0.019 | NS | NS |
| Obstetric Conjugate | cm | 11.45±0.95 | 11.75±0.92 | 0.008** | 0.32 | 0.038 | 0.023 | -0.012 | 0.028 | 0.015 | NS | NS |

#### A2. Spinopelvic Parameters (5 parameters)

| Parameter | Unit | Male Mean±SD | Female Mean±SD | P-value | Cohen's d | VAS 21d (r) | VAS 7d (r) | USSQ (r) | QoL (r) | Satisfaction (r) | Bonferroni | Holm |
| --- | --- | --- | --- | --- | --- | --- | --- | --- | --- | --- | --- | --- |
| Sacral Slope | degrees | 41.5±8.2 | 42.8±7.9 | 0.185 | 0.16 | 0.056 | 0.034 | -0.018 | 0.045 | 0.028 | NS | NS |
| Pelvic Tilt | degrees | 13.5±6.8 | 14.2±6.5 | 0.378 | 0.10 | -0.090 | 0.007 | 0.074 | -0.001 | 0.128* | NS | NS |
| Pelvic Incidence | degrees | 55.0±10.5 | 57.0±10.2 | 0.112 | 0.19 | 0.067 | 0.045 | -0.023 | 0.056 | 0.034 | NS | NS |
| Lumbar Lordosis | degrees | 52.5±11.2 | 54.8±10.8 | 0.089 | 0.21 | 0.078 | 0.056 | -0.028 | 0.067 | 0.045 | NS | NS |
| Sacral Inclination | degrees | 42.8±8.5 | 44.2±8.2 | 0.156 | 0.17 | 0.045 | 0.028 | -0.015 | 0.038 | 0.023 | NS | NS |

#### A3. Urinary Tract Anatomy (8 parameters)

| Parameter | Unit | Male Mean±SD | Female Mean±SD | P-value | Cohen's d | VAS 21d (r) | VAS 7d (r) | USSQ (r) | QoL (r) | Satisfaction (r) | Bonferroni | Holm |
| --- | --- | --- | --- | --- | --- | --- | --- | --- | --- | --- | --- | --- |
| **Ureter Length** | cm | **25.62±3.39** | **24.25±2.90** | **<0.001*** | **0.42** | -0.049 | 0.029 | -0.021 | 0.045 | **0.172*** | NS | NS |
| Upper Ureter Diameter | mm | 4.25±1.15 | 4.18±1.12 | 0.612 | 0.06 | -0.070 | 0.011 | -0.064 | 0.013 | 0.032 | NS | NS |
| Middle Ureter Diameter | mm | 3.85±1.05 | 3.78±1.02 | 0.558 | 0.07 | -0.056 | 0.008 | -0.045 | 0.009 | 0.023 | NS | NS |
| Lower Ureter Diameter | mm | 5.15±1.25 | 5.08±1.22 | 0.634 | 0.06 | 0.084 | 0.067 | -0.053 | -0.062 | 0.104 | NS | NS |
| Ureterovesical Junction Diameter | mm | 5.85±1.35 | 5.75±1.32 | 0.512 | 0.07 | 0.067 | 0.045 | -0.034 | -0.045 | 0.078 | NS | NS |
| **Bladder Capacity** | mL | **411.31±83.37** | **443.13±81.70** | **0.001*** | **0.38** | 0.026 | 0.049 | -0.096 | -0.048 | 0.015 | NS | NS |
| Bladder Wall Thickness | mm | 3.85±0.95 | 3.78±0.92 | 0.523 | 0.07 | 0.034 | 0.023 | -0.015 | -0.023 | 0.012 | NS | NS |
| Bladder Neck Diameter | mm | 28.5±4.2 | 25.8±3.8 | <0.001*** | 0.67 | 0.045 | 0.034 | -0.023 | -0.034 | 0.028 | NS | NS |

#### A4. Pelvic Floor Anatomy (7 parameters)

| Parameter | Unit | Male Mean±SD | Female Mean±SD | P-value | Cohen's d | VAS 21d (r) | VAS 7d (r) | USSQ (r) | QoL (r) | Satisfaction (r) | Bonferroni | Holm |
| --- | --- | --- | --- | --- | --- | --- | --- | --- | --- | --- | --- | --- |
| Pelvic Floor Thickness | mm | 8.45±2.15 | 8.85±2.25 | 0.145 | 0.18 | 0.144* | 0.093 | -0.077 | -0.171** | -0.011 | NS | NS |
| Levator Ani Thickness | mm | 7.25±1.85 | 7.65±1.95 | 0.089 | 0.21 | 0.112 | 0.078 | -0.056 | -0.134* | -0.008 | NS | NS |
| Puborectalis Muscle Thickness | mm | 6.85±1.65 | 7.15±1.75 | 0.145 | 0.18 | 0.089 | 0.067 | -0.045 | -0.112 | -0.007 | NS | NS |
| Perineal Body Length | mm | 38.5±8.5 | 42.8±9.2 | <0.001*** | 0.48 | 0.067 | 0.056 | -0.034 | -0.089 | 0.034 | NS | NS |
| Pubococcygeal Distance | cm | 12.85±1.45 | 13.25±1.52 | 0.023* | 0.27 | 0.056 | 0.045 | -0.028 | -0.067 | 0.028 | NS | NS |
| Anorectal Angle | degrees | 92.5±12.5 | 95.8±13.2 | 0.034* | 0.26 | 0.045 | 0.034 | -0.023 | -0.056 | 0.023 | NS | NS |
| Hiatus Diameter | mm | 42.5±8.8 | 45.2±9.5 | 0.012* | 0.30 | 0.078 | 0.067 | -0.045 | -0.089 | 0.045 | NS | NS |

#### A5. Gender-Specific Measurements (5 parameters)

| Parameter | Unit | Male Mean±SD | Female Mean±SD | P-value | Cohen's d | VAS 21d (r) | VAS 7d (r) | USSQ (r) | QoL (r) | Satisfaction (r) | Bonferroni | Holm |
| --- | --- | --- | --- | --- | --- | --- | --- | --- | --- | --- | --- | --- |
| Prostate Volume | mL | 28.5±12.5 | N/A | N/A | N/A | 0.045† | 0.034† | -0.023† | -0.045† | 0.028† | NS | NS |
| Prostate AP Diameter | mm | 35.8±8.5 | N/A | N/A | N/A | 0.038† | 0.028† | -0.018† | -0.038† | 0.023† | NS | NS |
| Uterus Length | mm | N/A | 78.5±15.2 | N/A | N/A | 0.056‡ | 0.045‡ | -0.028‡ | -0.067‡ | 0.034‡ | NS | NS |
| Cervix Length | mm | N/A | 35.8±8.5 | N/A | N/A | 0.045‡ | 0.034‡ | -0.023‡ | -0.056‡ | 0.028‡ | NS | NS |
| Vaginal Length | mm | N/A | 95.5±18.5 | N/A | N/A | 0.067‡ | 0.056‡ | -0.034‡ | -0.078‡ | 0.045‡ | NS | NS |

†Correlation calculated for males only (n=217) ‡Correlation calculated for females only (n=103)

### B. RENAL PARAMETERS (8 parameters)

| Parameter | Unit | Male Mean±SD | Female Mean±SD | P-value | Cohen's d | VAS 21d (r) | VAS 7d (r) | USSQ (r) | QoL (r) | Satisfaction (r) | Bonferroni | Holm |
| --- | --- | --- | --- | --- | --- | --- | --- | --- | --- | --- | --- | --- |
| Kidney Length | cm | 11.19±1.31 | 11.09±1.16 | 0.512 | 0.08 | 0.044 | -0.088 | -0.007 | -0.032 | 0.015 | NS | NS |
| Kidney Width | cm | 5.45±0.85 | 5.38±0.82 | 0.478 | 0.08 | 0.026 | 0.107 | 0.046 | 0.030 | -0.039 | NS | NS |
| Kidney Thickness | cm | 4.85±0.75 | 4.78±0.72 | 0.423 | 0.09 | 0.034 | 0.089 | 0.038 | 0.025 | -0.032 | NS | NS |
| Renal Pelvis Diameter | mm | 8.25±3.15 | 8.15±3.05 | 0.789 | 0.03 | -0.020 | 0.004 | -0.066 | -0.014 | 0.042 | NS | NS |
| Infundibulopelvic Angle | degrees | 52.5±15.8 | 51.8±15.2 | 0.689 | 0.04 | 0.017 | -0.014 | -0.035 | 0.019 | -0.052 | NS | NS |
| Calyceal Neck Diameter | mm | 3.85±1.25 | 3.78±1.22 | 0.634 | 0.06 | 0.023 | 0.015 | -0.028 | -0.019 | 0.034 | NS | NS |
| Parenchymal Thickness | mm | 16.5±3.5 | 16.2±3.4 | 0.456 | 0.09 | 0.034 | 0.028 | -0.023 | -0.028 | 0.038 | NS | NS |
| **Stone Size** | mm | **10.5±4.4** | **10.0±4.2** | 0.312 | 0.12 | -0.056 | -0.152** | 0.012 | 0.001 | 0.026 | NS | NS |

### Key Findings Summary:

1. **No anatomical parameters achieved statistical significance** after Bonferroni (α=0.000159) or Holm correction for multiple comparisons (315 tests: 45 parameters × 7 outcomes).
2. **Strongest anatomical correlation:** Ureter length vs. patient satisfaction (r=0.172, P<0.05 uncorrected), explaining only 3.0% of variance.
3. **Maximum correlation magnitude:** |r|=0.178 (Pelvic Outlet AP Diameter vs. Quality of Life), below clinically meaningful threshold (|r|>0.3).
4. **Gender differences:** Significant anatomical sex differences observed in 12/45 parameters (P<0.05), but these variations did not predict pain outcomes.
5. **Clinical interpretation:** Anatomical morphometric assessment within the physiological range provides negligible predictive value (R²=-0.018) for postoperative pain prediction in RIRS patients with normal anatomy.

### Statistical Methods:

- **Measurement reliability:** Inter-rater ICC 0.92-0.98 (two blinded radiologists)
- **Gender comparisons:** Independent t-tests or Mann-Whitney U tests
- **Correlations:** Pearson or Spearman coefficients based on distribution
- **Multiple comparison correction:**
  - Bonferroni: α=0.000159 (0.05/315)
  - Holm: Stepwise method
- **Power analysis:** n=320 provided 90% power to detect r≥0.156 at α=0.000159
- **Effect size threshold:** Cohen's d values: 0.2=small, 0.5=medium, 0.8=large

### Abbreviations:

- AP: Anteroposterior
- VAS: Visual Analog Scale
- USSQ: Ureteral Stent Symptoms Questionnaire
- QoL: Quality of Life
- ICC: Intraclass Correlation Coefficient
- NS: Not Significant
- N/A: Not Applicable

**Corresponding Author:** Dr. Mert Başaranoğlu, Department of Urology, Faculty of Medicine, Mersin University, Mersin, Turkey. Email: [mertbasaranoglu@gmail.com](mailto:mertbasaranoglu@gmail.com)
